# Metabolomic and Transcriptomic Comparison of Solid-State and Submerged Fermentation of *Penicillium expansum* KACC 40815

**DOI:** 10.1371/journal.pone.0149012

**Published:** 2016-02-10

**Authors:** Hyang Yeon Kim, Do Yeon Heo, Hye Min Park, Digar Singh, Choong Hwan Lee

**Affiliations:** Department of Bioscience and Biotechnology, Konkuk University, 120 Neungdong-ro, Gwangjin-gu, Seoul, 05029, Republic of Korea; Korea University, REPUBLIC OF KOREA

## Abstract

*Penicillium* spp. are known to harbor a wide array of secondary metabolites with cryptic bioactivities. However, the metabolomics of these species is not well-understood in terms of different fermentation models and conditions. The present study involved metabolomics profiling and transcriptomic analysis of *Penicillium expansum* 40815 under solid-state fermentation (SSF) and submerged fermentation (SmF). Metabolite profiling was carried out using ultra-performance liquid chromatography quadruple time-of-flight mass spectrometry with multivariate analysis, followed by transcriptomic analyses of differentially expressed genes. In principal component analysis, the metabolite profiling data was studied under different experimental sets, including SSF and SmF. The significantly different metabolites such as polyketide metabolites (agonodepside B, rotiorin, verrucosidin, and ochrephilone) and corresponding gene transcripts (polyketide synthase, aromatic prenyltransferase, and terpenoid synthase) were primarily detected under SmF conditions. In contrast, the meroterpenoid compounds (andrastin A and C) and their genes transcripts were exclusively detected under SSF conditions. We demonstrated that the metabolite production and its corresponding gene expression levels in *P*. *expansum* 40815 were significantly influenced by the varying growth parameters and the immediate environment. This study further provides a foundation to produce specific metabolites by regulating fermentation conditions.

## Introduction

*Penicillium* spp. are ubiquitously distributed filamentous fungi with antimicrobial metabolites that are of unparalleled importance to the pharmaceutical and food industries [1]. One of the most widely studied species, *Penicillium expansum*, is known to produce an array of cryptic metabolites with a spectrum of bioactivities, including anti-parasitic, immunosuppressive, antioxidant, antifungal, and antibacterial effects [2, 3]. In our previous study, we examined four different *Penicillium* spp. for the production of antioxidant metabolites and found that *P*. *expansum* exhibited highest radical scavenging activities [4]. Recent studies have further demonstrated the indispensable commercial applications of *Penicillium* extrolites in the pharmaceutical and food industries [5, 6]. The stringent mechanisms of secondary metabolite biosynthesis in filamentous fungi often limit their substantial production under laboratory conditions. The fermentative production of these metabolites can be maximized by optimizing the various screening and culture conditions, such as media composition, incubation period, pH, and temperature [7–9]. Moreover, the manipulation of culture conditions can also trigger the required alterations in fungal morphology (hyphal, pelleted, or homogenous), growth kinetics, physiology, and chemical diversities, *i*.*e*., production of secondary metabolites [10, 11]. Thus, examination of the optimal culture conditions and fermentation states, including submerged fermentation (SmF) or solid-state fermentation (SSF), facilitates optimization of the production kinetics and fungal metabolomics. SmF involves cultivation in homogenous aqueous growth medium, while SSF involves culture growth on solid surfaces or particles. However, statistical analyses of the total metabolic changes according to the fermentation state in *Penicillium* spp. has not been reported. Therefore, a comprehensive study involving the comparative analysis of metabolite production profiles under different fermentation states can help to identify and overcome bottlenecks in the metabolomic outputs of *Penicillium* spp.

Metabolomics is the unbiased global study of the entire complement of metabolites in a biological sample under a given set of conditions, serving as an instrument to determine the vital phenomena in a cell, tissue, organ, or whole organism [12, 13]. Metabolomics, which is the final version of the ‘omics cascade’, represents the final step in an organism’s phenotype, and thus can address the quantitative expression of each metabolite in a sample without bias [14]. In recent years, mass spectrometry (MS) has emerged as an important tool in metabolomics studies because of its high precision in qualitative and quantitative detection of an array of trace metabolites in a biological sample [9, 15, 16]. MS-based metabolomics profiles are further subjected to statistical multivariate analysis, such as principal component analysis (PCA), to determine the effects of environmental stimuli (growth or stress conditions) on an organism’s metabolome. However, a more elaborate approach may involve the integration of metabolomics profile with their corresponding genes and quantifying their regulatory properties in the host genome. Here, the correlation between the two sets of ‘omics’ data, metabolomics and transcriptomics, can give us the opportunity to understand an organisms’ metabolic phenotype and its underlying mechanisms of gene regulation under different physiological states and growth conditions [17]. A holistic assessment of the metabolome that includes transcriptome bias, influenced by the two different fermentation states of *Penicillium* spp., offers insight into global and targeted metabolite profiling [18]. The approach will complement the understanding of the chemotaxonomy, metabolite fingerprinting, and transcript differences of different organisms under a variety of growth conditions.

In this study, we report the effects of different fermentation conditions on the non-targeted metabolome of *P*. *expansum* 40815. The extracted metabolites were analyzed using ultra-performance liquid chromatography-quadrupole-time of flight-MS (UPLC-Q-TOF-MS), followed by liquid chromatography-electro spray ionization-MS (LC-ESI-MS) validation and multivariate statistical data analysis. We aimed to determine the underlying transcriptomics to correlate the differential expression of corresponding genes under varying fermentation conditions for *Penicillium* culture. This study establishes a fermentation model for differential metabolome and transcriptome expression for *Penicillium* spp. that can be used in in scale-up studies.

## Materials and Methods

### Chemicals and Culture Media

Water, methanol, ethyl acetate, and acetonitrile were purchased from Fisher Scientific (Pittsburgh, PA, USA). Malt extract broth (MEB) and agar (MEA) were purchased from Becton Dickinson (Franklin Lakes, NJ, USA). Analytical-grade formic acid was purchased from Sigma Aldrich (St. Louis, MO, USA).

### Fungal Strains and Preparation of Seed Cultures

The *P*. *expansum* KACC 40815 used in the study was obtained from the Korean Agricultural Culture Collection (KACC, Suwon, South Korea). The strain was transferred from -80°C frozen stocks to MEA plates for pre-culture, prior to using the cells as an inoculum. The frozen stocks were grown on MEA plates for 7 days at 28°C in the dark and then the spores were harvested and stored in 20% glycerol. The *Penicillium* strain was first cultured on MEA plates for 3 days, and then inoculated onto 6-mm discs on fresh MEA and MEB for the SSF and SmF procedures, respectively.

### Culture Conditions

For liquid culture (SmF), 50 mL of MEB medium was culture inoculated into 250-mL Erlenmeyer flasks prepared in triplicate and incubated at 28°C with agitation at 200 rpm. For SSF, inoculated MEA plates were maintained at 28°C under dark conditions. The *Penicillium* cultures in the two different fermentation states (SSF and SmF) were harvested from 4–16 days at 2-day intervals and extracted for metabolite profiling. All samples were prepared in triplicate.

### Extraction of Fungal Metabolites

The SSF culture extracts of *P*. *expansum* were prepared by adding 20 mL of ethyl acetate to the whole chopped agar plates, followed by vigorous mixing in a rotary shaker at 200 rpm for 24 h. Simultaneously, the culture supernatant from the SmF state was filtered using 7-μm filter paper, while the residue pellets were further extracted using 20 mL ethyl acetate in the rotary shaker at 200 rpm for 24 h. The filtrate supernatants were solvent-partitioned (liquid-liquid extracted) using 1:1 ethyl acetate and then mixed with the pellet extracts. The extracts were dried by evaporation, dissolved in 2 mL of methanol, and then filtered through a disposable 0.45-μm polytetrafluoroethylene filter membrane for further analyses.

### UPLC-Q-TOF-MS Analysis

UPLC-Q-TOF-MS analysis was performed using a Waters Micromass Q-TOF Premier with UPLC Acquity system^™^ (Waters, Milford, MA, USA). The fermentation extracts were analyzed using a C18 column (100 × 2.1 mm, 1.7 μm) and Acquity UPLC BEH (Waters). The mobile phase consisted of water and acetonitrile containing 0.1% formic acid (*v*/*v*). The initial gradient condition of the mobile phase was maintained at 0% acetonitrile for 0.3 min, and then gradually increased to 30% acetonitrile over 3 min, 40% for 1 min, and 100% for 8 min. The acetonitrile concentration was then maintained at 100% for 2 min and decreased to 0% for 2 min. The flow rate was maintained at 0.3 mL/min and 5 μL of sample was injected. ESI analysis was performed in the both negative (ESI-) and positive (ESI+) mode within the range of *m/z* 100–1000. The operating parameters were as follows: ion source temperature 200°C, cone gas flow 50 L/h, desolvation gas flow 600 L/h, capillary voltage 2.8 kV, and cone voltage up to 35 V with 10,000 resolution. Each sample (24 samples) obtained from SSF and SmF condition were analyzed in triplicate for instrumental analysis.

### LC-ESI-MS Analysis

Further analysis and validation of the selected metabolites were performed using a liquid chromatography ESI trap MS (LC-ESI-IT-MS) system. Liquid chromatographic analysis was performed on a Varian 500MS ion trap mass spectrometer (Varian Inc., Palo Alto, CA, USA), which consisted of an LC pump (Varian 212), a photodiode array detector (ProStar 335, Agilent Technologies, Santa Clara, CA, USA), and an auto sampler (ProStar 410). The LC system was equipped with a C18 column (100 × 2.0 mm, 3 μm) (Varian). The binary mobile phase consisted of water and acetonitrile containing 0.1% formic acid (*v*/*v*). The initial gradient condition of the mobile phase was 10% acetonitrile for 2 min, gradually increasing acetonitrile to 100% over 28 min. The 100% acetonitrile condition was maintained for 5 min and then acetonitrile was sharply reduced to 10% over 0.06 min and maintained for 5 min. Next, 10 μL of the extract samples were injected and the flow rate was maintained at 0.2 mL/min. ESI-MS was performed in the positive and negative modes over the range of *m/z* 100–1000. The running parameters were as follows: drying temperature 350°C; needle voltage 5 kV; capillary voltage 70 V, drying gas pressure (nitrogen) 10 psi; nebulizer gas pressure (air) 35 psi. MS^n^ analysis was performed using scan type turbo data dependent scanning under the same conditions used for positive and negative mode MS scanning.

### Data Processing and Multivariate Analysis

UPLC-Q-TOF-MS data were acquired using MassLynx software (version 4.1; Waters) and raw data files were converted into NetCDF format (*.cdf) using the MassLynx DataBridge (version 4.1; Waters). After conversion, peak detection, retention time correction, and alignment were automatically conducted using the Metalign software package (http://www.metalign.nl). The MetAlign parameters were set as follows: peak slope factor of 2.0, peak threshold factor of 2.0, peak threshold of 20 and average peak width at half height of 10, which corresponds to a retention time of 0 to 10 min, and mass range of 100 to 1,000 for UPLC-Q-TOF-MS. The resulting *.csv file contained 5,493 variables of the nominal mass peak intensity data with retention time, and the value was transferred to an excel data sheet for multivariate analysis.

Statistical analysis was performed using SIMCA-P+ (version 12.0, Umetrics, Umea, Sweden) for PCA and orthogonal partial least squares-discriminant analysis (OPLS-DA). All variables were pareto-scaled and log_10_-transformed mean-centered for statistical analysis. The unsupervised PCA was used to observe the distribution of *Penicillium* metabolites produced by SmF and SSF. The two fermentation groups were separated by OPLS-DA and their potential variables were selected based on the variable importance in projection (VIP > 1.0) values and *p*-value (*p* < 0.05). The *p*-value of each metabolite for the different methods was determined using Statistica 7 (StatSoft Inc., Tulsa, OK, USA). Significantly different secondary metabolites were confirmed using the UPLC-Q-TOF-MS chromatogram.

### RNA Sequence Analysis

#### Sequencing

For RNA sequencing, 0-, 6-, and 14-day culture replicates from SSF and SmF were harvested and washed twice with phosphate-buffered saline. The library was prepared from 1 μg of total RNA from cell pellet using the Truseq RNA Sample Prep Kit v2 (Illumina, Inc., San Diego, CA, USA). mRNA was isolated from total RNA using poly-T oligo-attached magnetic beads at an incubation temperature of 94°C for 8 min, producing mRNA fragments of 150 base pairs (bp). Blunt-end phosphorylated cDNA was synthesized from mRNA fragments using random primers in a reverse transcriptase reaction. The poly “A” tail was linked at 3′ end, followed by ligation of a 3′ sequencing adaptor. The RNA-seq library was PCR-amplified, quality-checked using a QC-BioAnalyzer, and pooled at similar concentrations and molecular sizes. Finally, high-throughput paired-end 100-bp sequencing was performed using the Illumina Hiseq 2500.

#### Sequence Read Mapping on the Reference Genome and the Identification of DEGs Using Group Variables

The RNA-seq reads were mapped against the reference genome (*Penicillium expansum* ATCC 24692 v1.0 from JGI) using Bowtie2, included in TopHat (v2.0.9); a fast spliced junction mapper software program for RNA-seq reads against the reference genome. The aligned result from the TopHat in the BAM (.bam) file format was then analyzed using Cuffdiff (v2.2.0), and differentially expressed gene (DEG) transcripts were identified. The data for RNA-seq DEGs were normalized using the fragments per kilobase per million mapped reads method. Cuffdiff provides various output files, and using one of its outputs, “gene_exp.diff”, DEGs were identified. Based on the IPR database, we compared the differential transcriptomics between SSF and SmF cultures of the *Penicillium* strain.

## Results

### PCA for Non-targeted Metabolomics Data from *P*. *expansum* 40815 in SSF and SmF

**Fig 1:**
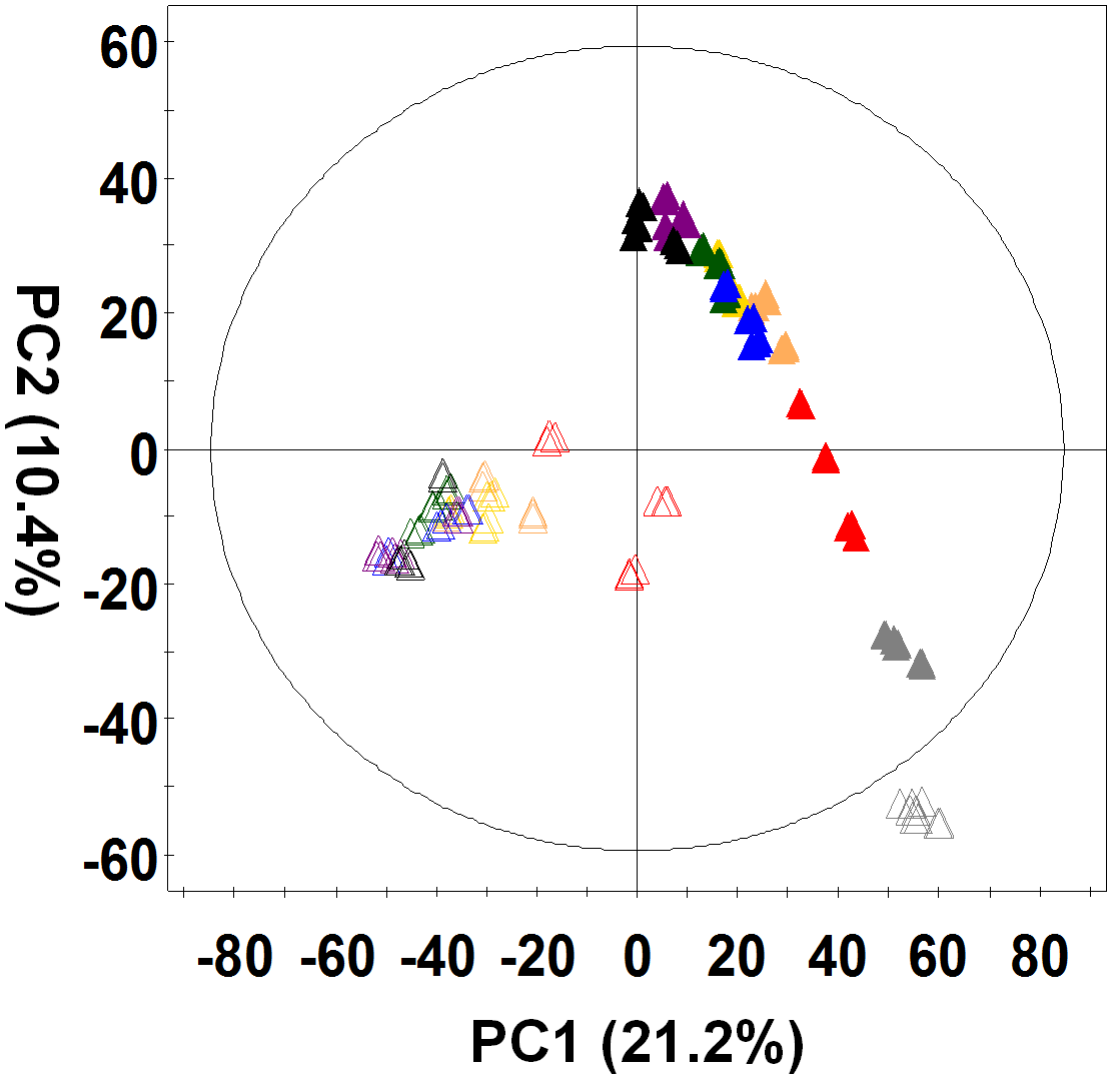
Principal component analysis (PCA) score plot derived from the UPLC-Q-TOF-MS dataset of *P*. *expansum* 40815 during fermentation. (△: Submerged fermentation, ▲: Solid-state fermentation, gray: 0 days, red: 4 days, orange: 6 days, yellow: 8 days, green: 10 days, blue: 12 days, violet: 14 days, black: 16 days)

UPLC-Q-TOF-MS data in positive mode for *Penicillium* extracts from SSF and SmF fermentation states were subjected to multivariate analysis using PCA because we detected more peaks and available areas in the positive ion mode than those in the negative mode. PCA showed the variance explained with 16 principal components. Among them, the scores of the first principal component (PC1) accounting for the greatest possible variance in the data set and the following component (PC2) were 21.2% and 10.4%. The total sum of each principal components were 70% over. After 4 days of fermentation, the *P*. *expansum* extracts from SmF and SSF were divided into two groups by PC1. The extracts of SmF and SSF were further divided by PC2 after 16 days of fermentation (Fig 1). The chromatograms for SmF and SSF during fermentation in the positive mode are presented in S1 Fig. The 0 day chromatograms for SmF and SSF were similar; however, the chromatograms differed for SmF and SSF after 4 days of fermentation.

### Metabolome-Transcriptome Variations of *P*. *expansum* 40815 in SSF and SmF

#### Metabolomics Variations for Secondary Metabolite Production

**Fig 2:**
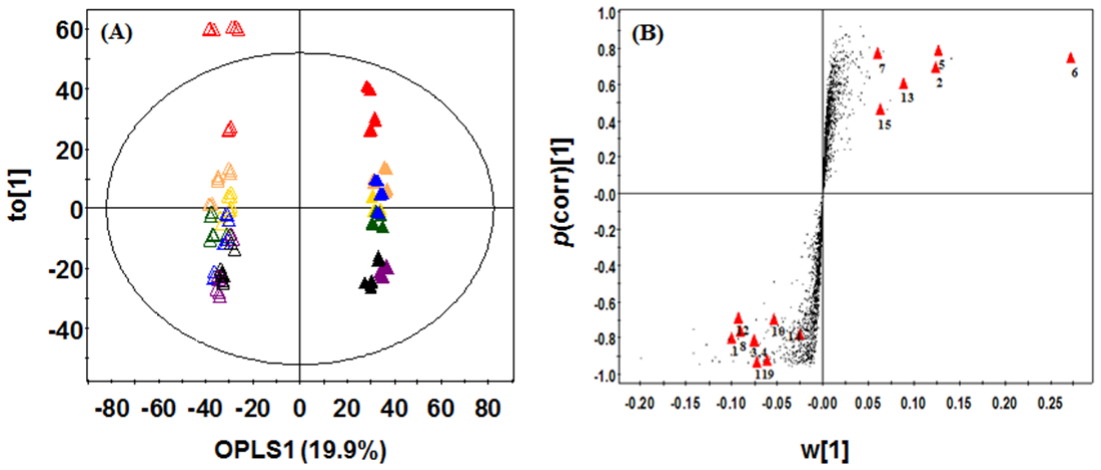
(A) Orthogonal partial least squares-discriminant analysis (OPLS-DA) score plot and (B) loading S-plots of different cultures in the UPLC-Q-TOF-MS based dataset. The significantly different metabolites (▲) according to different fermentation condition are highlighted in the S-plot. The numbering of metabolites is identical to that in Table 1. (△: Submerged fermentation, ▲: Solid-state fermentation, red: 4 days, orange: 6 days, yellow: 8 days, green: 10 days, blue: 12 days, violet: 14 days, black: 16 days. 1: N.I.1, 2: N.I.2, 3: N.I.3, 4:: N.I.4, 5: Agonodepside B, 6: Rotiorin, 7: Verrucosidin, 8: N.I.5, 9: Andrastin A, 10: N.I.6, 11: N.I.7, 12: N.I.8, 13: Ochrephilone, 14: Andrastin C, 15: N.I.9)

**Fig 3:**
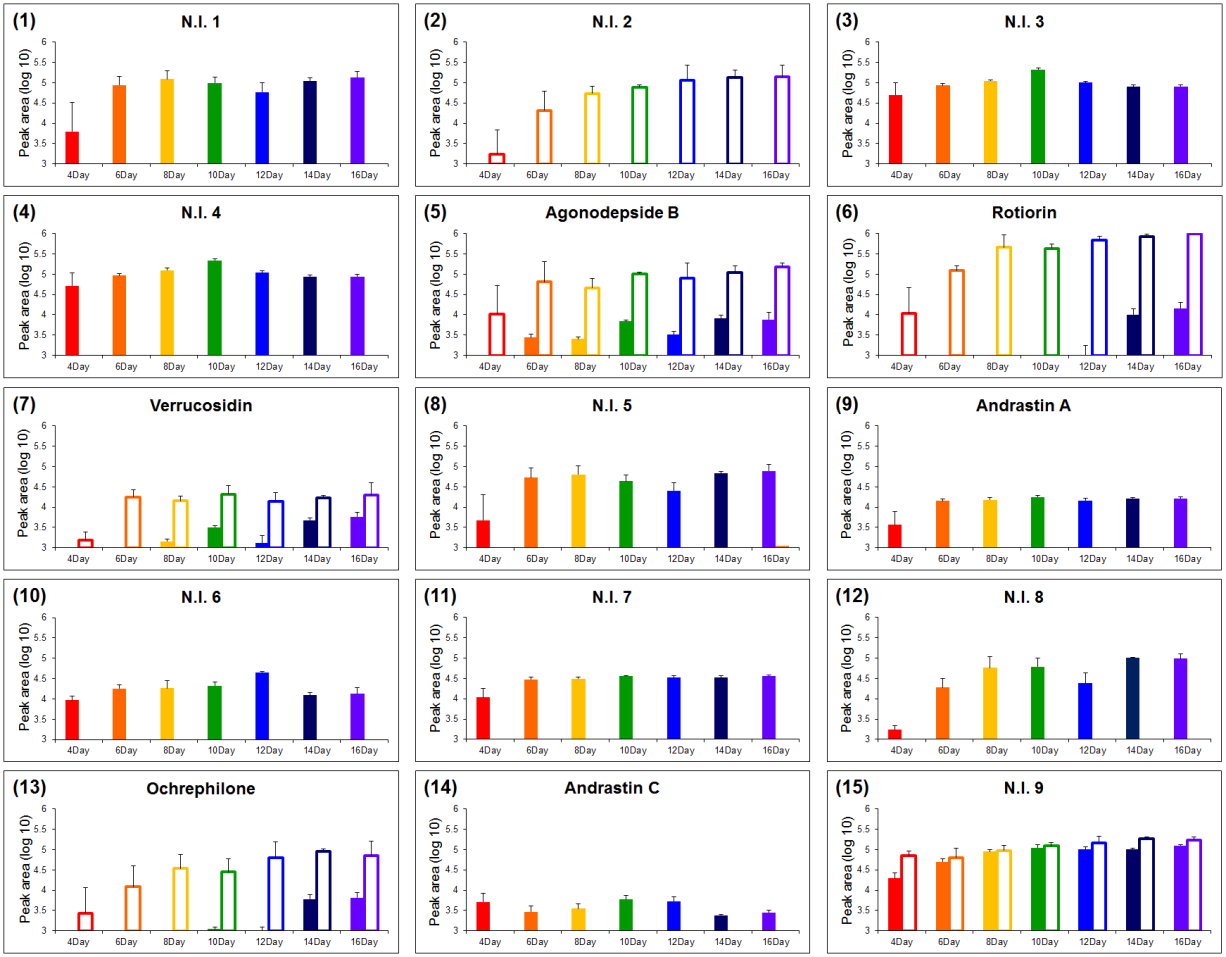
Production profiles of variant metabolites from solid-state and submerged fermentation of *P*. *expansum* 40815. The Y-axis of graphs indicates the UPLC-Q-TOF-MS data transformed by log10. (△: Submerged fermentation, ▲: Solid-state fermentation; red: 4 days, orange: 6 days, yellow: 8 days, green: 10 days, blue: 12 days, navy: 14 days, violet: 16 days)

**Fig 4:**
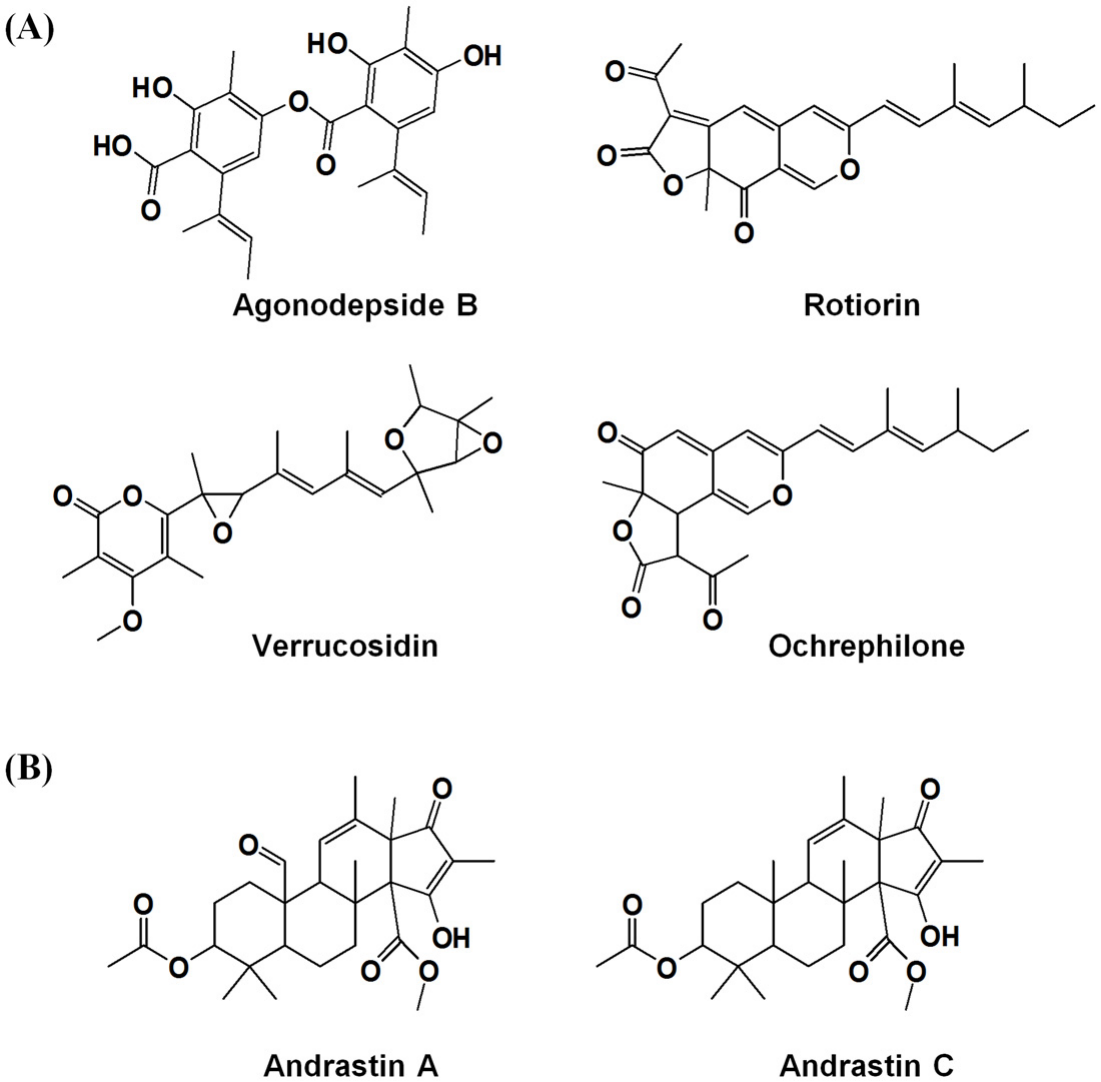
Structures of variant metabolites significantly produced in (A) submerged and (B) solid-state fermentation.

To select the differentially expressed variant metabolites between SSF and SmF, the OPLS-DA model was applied using UPLC-Q-TOF-MS data set in positive ion mode and showed the separation with component 1 (19.9%) (Fig 2A). And also, we obtained the significance with *p*-value (below 0.001) in this model. We selected 15 variables by VIP (> 1.0) and *p*-value (< 0.05), and graphed significantly different metabolites on an S-plot (Fig 2B). These compounds were tentatively identified by matching the molecular weight, retention time, UV spectra, MS^n^ fragmentation pattern, and high resolution mass data (Q-TOF-MS) with the Dictionary of Natural Products 2008 (Taylor & Francis, Boca Raton, FL, USA), Antibase 3.0 (CambridgeSoft Corporation, Cambridge, MA, USA), and published literatures (Table 1). The MS^n^ fragmentation pattern of each metabolite was compared with the simulated character of fragmentation using Mass frontier software 4.0 (Highchem, Bratislava, Slovakia). The isotope patterns and exact mass of the metabolites were compared with the recommended chemical composition based on i-FIT value (<2.0) and mDa (<5.0), respectively. We putatively identified six metabolites, including agonodepside B, rotiorin, verrucosidin, ochrephilone, and andrastin A and C. Of the six metabolites, agonodepside B, rotiorin, verrucosidin, and ochrephilone are polyketide compounds, while andrastin A and C are meroterpenoids. To determine metabolites production by SmF and SSF, we examined the logarithmic intensities from UPLC-Q-TOF-MS data for both of the fermentation states (Fig 3). Of the 15 selected metabolites, 5 metabolites, including agonodepside B, rotiorin, verrucosidin, ochrephilone, and one non-identified metabolite (N.I.9) were produced in both SmF and SSF. However, the levels of these 5 compounds were higher in SmF than in SSF. One non-identified metabolite (N.I.2) was only detected in the extract of SmF, while andrastin A and C and 7 non—identified metabolites (N.I.1, 3, 4, 5, 6, 7 and 8) were only detected in the SSF extract. The structures of the identified compounds are presented in Fig 4.

**Table 1:** Putative identified metabolites from the *Penicillium expansum* 40815 according to different fermentation condition on the basis of UPLC-QTOF-MS and LC-ESI-MS data.

No.	Putative identification	t_R_^a^ (min)	UPLC-Q-TOF-MS						LC-ESI-MS		*p*-value^d^
			[M-H]^-^	[M+H]^+^	M.W.^b^	Error (mDa)	i-Fit (norm)	Exact mass ([M-H]^-^)	MS^n^ fragment ions ^c^	UV λmax (nm)	
1	N.I.1	5.67	609.2685	611.2850	610	-	-	-	611>539, 356, 355>493, 479, 424, 411, 491, 450, 410, 285	206, 219, 227, 233, 308	7.95E-27
2	N.I.2	5.80	375.1729	399.1820	376	-	-	-	399>381>348	244, 276, 301, 382	6.77E-20
3	N.I.3	6.26	-	385.1960		-	-	-	-	-	5.40E-30
4	N.I.4	6.29	-	457.2550		-	-	-	-	-	6.53E-30
5	Agonodepside B	6.32	425.1614	427.1780	426	-2.3	0.6	427.1757	427>381>338	244, 272, 311	1.85E-26
6	Rotiorin	6.38	379.1558	381.1673	380	2.9	1.4	381.1702	381>363, 348>347, 333, 318, 334	244, 275, 307, 384	1.38E-24
7	Verrucosidin	6.74	415.2052	417.2260	416	1.7	0.6	414.2277	417>381, 353, 363, 339	246, 273, 299	4.46E-24
8	N.I.5	6.75	661.3013	663.3160	662	-	-	-	663>591>497, 353, 463, 478, 480, 495	210, 212, 218, 244	3.20E-23
9	Andrastin A	7.59	485.2540	487.2670	486	2.6	0.1	487.2696	509[M+Na]+>437>343, 311, 344	201, 207, 214, 219	2.07E-50
10	N.I.6	7.89	-	361.1580	-	-	-	-	361>293>245	-	4.57E-19
11	N.I.7	8.75	863.5703	887.5706	864	-	-	-	887[M+Na]+>746, 747>633, 520, 560, 447, 521, 634, 661	214, 219, 234, 235	8.78E-53
12	N.I.8	8.77	-	498.3740	-	-	-	-	498>263	-	2.18E-17
13	Ochrephilone	8.98	381.1641	383.1838	382	1.8	0.5	383.1856	383>367, 312, 365>336, 335, 295, 281, 297, 257, 282, 296	244, 302, 412	3.73E-15
14	Andrastin C	9.00	471.2719	473.2930	472	-2.7	1.7	473.2903	-	202, 214, 227, 245	1.48E-29
15	N.I.9	9.20	456.2734	458.2890	457	-	-	-	458>414, 430, 396>243, 340, 386, 187, 396	245, 285, 302	7.17E-09

^a^ Retention time,

^b^ Molecular weight,

^c^ Positive ion mode,

^d^ Indicated statistically significant difference by *t*-test

### Transcriptomic Variations in Secondary Metabolite Expression

**Fig 5:**
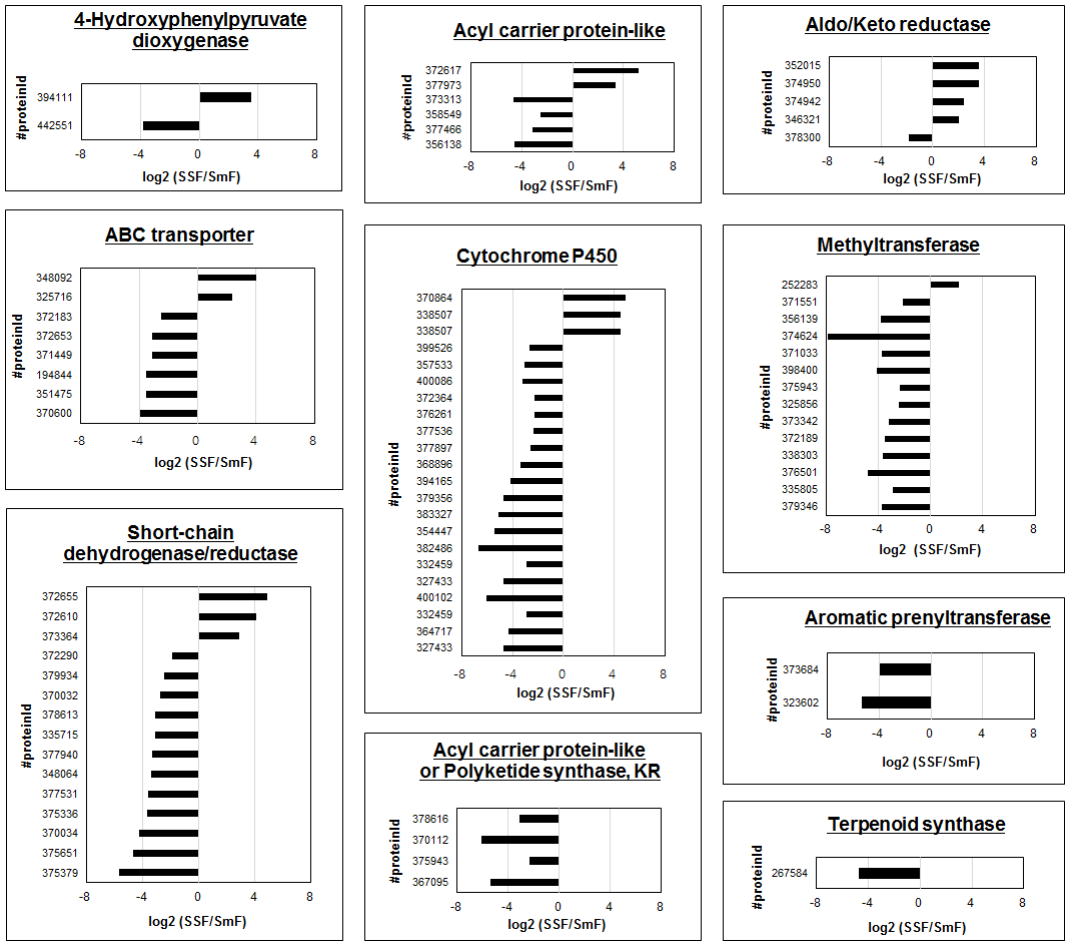
Transcript abundance values of DEGs in secondary metabolism. Abundance values (log2 SSF/SmF) and transcripts with significant variations (log2 SSF/SmF abundance greater than 1) are shown.

To investigate the relationship between the selected secondary metabolites and their gene transcriptomics, DEGs in SSF and SmF were identified. A total of 901 genes were significantly expressed, and 339 and 562 genes were up-regulated in SSF and SmF, respectively. Among the different expressed transcripts, 79 transcripts encoded proteins involved in secondary metabolite-related metabolism and showed differences in SSF and SmF (Fig 5). These transcripts included ABC transporter, acyl carrier protein, acyl or *O*-methyltransferase, cytochrome P450, aldo/keto reductase, short-chain dehydrogenase/reductase, aromatic prenyltransferase, terpenoid synthase, and polyketide synthase. Among these, ABC transporter, acyl carrier protein, acyl or *O*-methyltransferase, cytochrome P450, aldo/keto reductase, and short-chain dehydrogenase/reductase were highly expressed in both fermentation states. Polyketide synthase (or acyl carrier protein-like), aromatic prenyltransferase, and terpenoid synthase were highly expressed in SmF.

To identify transcripts related to polyketide and meroterpenoid synthesis, which are necessary for the synthesis of agonodepside B, rotiorin, verrucosidin, ochrephilone, and andrastin A and C, we compared each incubation time and state. We found 3 transcripts encoding terpenoid cyclase/protein prenyltransferase alpha-alpha toroid, terpenoid synthase, and polyketide synthase (Table 2). Terpenoid cyclases/protein prenyltransferase alpha-alpha toroid was only expressed on days 6 and 14 of SSF. Both terpenoid synthase and polyketide synthase were expressed in both fermentation states for different incubation times.

**Table 2:** Transcripts related with polyketide and meroterpenoid compounds synthesis in the different fermentation states and time.

Function	Protein ID	Ipr ID	Ipr Desc	Sample 1	Sample 2	Log2 (Sample 2/Sample 1)	*p*-value ^a^
Meroterpenoid synthesis	356228	IPR008930	Terpenoid cyclases/protein prenyltransferase alpha-alpha toroid	SSF 0 day	SSF 6 day *	Only expressed in 6 day	0.0067
Meroterpenoid synthesis	356228	IPR008930	Terpenoid cyclases/protein prenyltransferase alpha-alpha toroid	SSF 0 day	SSF 14 day *	Only expressed in 14 day	0.0064
Meroterpenoid synthesis	398546	IPR008949	Terpenoid synthase	SmF 0 day *	SSF 0 day	-2.56025	0.0005
Meroterpenoid synthesis	267584	IPR008949	Terpenoid synthase	SmF 6 day *	SSF 6 day	-5.75427	0.0197
Meroterpenoid synthesis	381859	IPR008949	Terpenoid synthase	SmF 6 day	SSF 6 day *	Only expressed in solid 6 day	0.0035
Meroterpenoid synthesis	267584	IPR008949	Terpenoid synthase	SmF 14 day *	SSF 14 day	-4.14505	0.0490
Polyketide synthesis	362066	IPR013968	Polyketide synthase, KR	SSF 0 day *	SSF 14 day	-4.20531	0.0414
Polyketide synthesis	371845	IPR013968	Polyketide synthase, KR	SSF 0 day *	SSF 14 day	-5.47295	0.0336
Polyketide synthesis	367095	IPR013968	Polyketide synthase, KR	SmF 0 day	SmF 6 day *	8.12118	0.0208
Polyketide synthesis	371845	IPR013968	Polyketide synthase, KR	SmF 0 day *	SmF 6 day	-4.36915	0.0010
Polyketide synthesis	375943	IPR013968	Polyketide synthase, KR	SmF 0 day *	SmF 6 day	-4.00845	0.0087
Polyketide synthesis	378616	IPR013968	Polyketide synthase, KR	SmF 0 day	SmF 6 day *	2.62765	0.0378
Polyketide synthesis	367095	IPR013968	Polyketide synthase, KR	SmF 0 day	SmF 14 day *	6.39115	0.0199
Polyketide synthesis	370112	IPR013968	Polyketide synthase, KR	SmF 0 day	SmF 14 day *	8.39086	0.0286
Polyketide synthesis	371845	IPR013968	Polyketide synthase, KR	SmF 0 day	SmF 14 day *	-5.97088	0.0006
Polyketide synthesis	375904	IPR013968	Polyketide synthase, KR	SmF 0 day	SmF 14 day *	2.73769	0.0328
Polyketide synthesis	375943	IPR013968	Polyketide synthase, KR	SmF 0 day	SmF 14 day *	-3.93090	0.0058
Polyketide synthesis	378616	IPR013968	Polyketide synthase, KR	SmF 0 day	SmF 14 day *	4.26984	0.0480
Polyketide synthesis	379126	IPR013968	Polyketide synthase, KR	SmF 0 day	SmF 14 day *	2.93721	0.0380
Polyketide synthesis	375943	IPR013968	Polyketide synthase, KR	SmF 0 day	SSF 0 day	-4.08802	5.0E-05
Polyketide synthesis	375904	IPR013968	Polyketide synthase, KR	SmF 6 day *	SSF 6 day	-6.51021	0.0439
Polyketide synthesis	370112	IPR013968	Polyketide synthase, KR	SmF 14 day *	SSF 14 day	-8.08628	0.0238

^a^ Indicated statistically significant difference by *t*-test

* Higher expressed transcripts than the other sample.

Moreover, ROS/defense-related enzymes including catalase and manganese- and iron-superoxide dismutase were relatively upregulated in SmF, while chloroperoxidase and DyP-type peroxidase were found with higher levels in SSF (S2 Fig).

## Discussion

We compared metabolites difference between different fermentation conditions, SSF and SmF, in *P*. *expansum* using metabolite profiles, and found the transcripts closely related with these metabolites through RNA sequence analysis. From fermentation techniques, SSF is best suited for fungi and microorganisms that require less moisture content, while SmF is best suited for microorganisms including bacteria that demand high moisture content [19]. The comparative studies on the enzyme production such as lipase, pectate lyase and α-amylase in *Penicillium* species between SSF and SmF have been well reported [20–22]. Moreover, production of secondary metabolites also depend on these fermentation states, which affect the difference of fermentation products. Present study showed some specific metabolites were produced according to fermentation states; Andrastin A and C in SSF-specific metabolites, agonodepside B, rotiorin, verrucosidin and ochrephilone in SmF-predominant metabolites. Among SmF-predominant metabolites, rotiorin and ochrephilone are in the azaphilones class, which are well-known as pigments of *Penicillium* species. Moreover, ochrephilone is a compound derived from rotiorin and is typically detected in *P*. *citreonigrum* [23, 24]. Agonodepside B and verrucosidin are depside and tremorgen compounds, respectively, and have also been detected in *Penicillium* species [25–28]. These 4 metabolites together comprise a class of polyketide compounds that were differentially up-regulated in SmF compared to in SSF, indicating the effects of different physiological states and growth conditions on polyketide biosynthetic pathways in *Penicillium* spp. (Fig 5). The series of polyketides are well-known for their anti-bacterial and anti-fungal activities, and *Penicillium* species have been reported to produce numerous polyketides with significant antimicrobial bioactivity [24].

In SSF-specific metabolites, andrastin A and C comprise class of meroterpenoid compounds isolated from *P*. *expansum* [29], which possess strong antitumor and proteolytic activities [30]. These compounds are derived from a 3,5-dimethylorsellinate precursor, and pass through alkylated polyketide and terpenoid intermediates by polyketide synthase-, methyl transferase-, terpenoid cyclase-, and ketoreductase-mediated biosynthetic pathways (S3 Fig). These enzyme transcripts were mainly observed in SSF, particularly terpenoid cyclase, which was expressed only on days 6 and 14, representing the key enzyme in meroterpenoid biosynthesis (Table 2). Rodriquez-Urra et al. [31] reported that sporulation is induced by signal metabolites, including dehydroaustinol and diorcinol, a class of meroterpenoids produced during SSF of *Aspergillus nidulans*. These results support that andrastin A and C (meroterpenoids) may act as signal factors for sporulation in SSF, explaining why these compounds were specifically detected in SSF. In addition, we observed a larger number of significantly variant metabolites in SSF, indicating its comparatively higher metabolic diversity compared to in SmF [32–34]. However, it is insufficient to fully explain the relationship between the specific- or predominant-metabolites and fermentation condition in *Penicillium* culture because of 9 non-identified metabolites. Further studies on the non-identified metabolites are needed to reveal the functions or the roles according to fermentation states in *Penicillium* spp. in depth. Although we did not completely identify some metabolites discriminated between SSF and SmF culture in *P*. *expansum*, these results demonstrate metabolites production and its related gene expression which are associated with secondary metabolism for *P*. *expansum* are largely affected by fermentation conditions.

Together with transcripts associated with polyketide and meroterpenoid synthesis, we found the expression ratio difference from ROS-related genes between SSF and SmF (S2 Fig). Different incubation conditions can lead different growth forms, such as filamentous and pelleted forms, which show distinct characteristics and can influence the mass transfer rate [35, 36]. In SSF, growth seems to be restricted by the solid matrix surface and limited oxygen transfer, while fungal cultures in SmF are exposed to vital hydrodynamic forces derived from aeration and vigorous agitation. This intense aeration often causes reactive oxygen species (ROS) generation in SmF, which has been linked with damage to genomic material, resulting in different transcriptomes and varying metabolite production [10]. In the transcriptomic analysis, ROS-related enzymes [37] such as catalase and manganese- and iron-superoxide dismutase were found to be differentially upregulated in SmF, while peroxidases were expressed at higher levels in SSF (S2 Fig). Moreover, glutathione *S*-transferase, a defense protein in plant cells [38], was highly expressed in SmF, which may be attributed to the agitation and aeration-induced stress conditions. In this study, we found that ROS-related enzymes were differentially expressed in the two culture systems, but their relationship remains unclear.

In this study, we comparatively evaluated the non-targeted metabolomic and targeted transcriptomic profiles of *P*. *expansum* 40815 under different fermentation states (SSF and SmF) and successfully annotated their significant metabolite and transcript variants. The production of different metabolites appears to be correlated with DEGs and the underlying biosynthetic pathways. These results further validate the empirical findings and recent reports suggesting the metabolite diversity among the *Penicillium* spp. in different ecological states, physiologies, and growth conditions. The variation in metabolite production and the altered expression levels of the corresponding genes provide a foundation for engineering specific fermentation conditions for specific metabolic outcomes. Our results present a holistic research platform with applications in industrial scale-up fermentation designs, as well as in studies involving chemodiversity or chemical ecology.

## Supporting Information

S1 Fig Chromatogram of *P*. *expansum* 40815 in SmF and SSF by UPLC-Q-TOF-MS analysis in positive mode. SmF: Submerged fermentation, SSF: Solid-state fermentation.
(PPTX)

S2 Fig Differentially expressed ROS/defense-related genes in SSF and SmF. Abundance values (log_2_ SSF/SmF) and transcripts with significant variations (log_2_ SSF/SmF abundance greater than 1) are shown.
(PPTX)

S3 Fig Biosynthesis pathway of andrastin (A) and azaphilones (B) series. (PPTX)

S1 File Raw file (*xlsx) for metabolite profiles of *P*. *expansum* 40815 in SmF and SSF by UPLC-Q-TOF-MS analysis in positive mode. SmF: Submerged fermentation, SSF: Solid-state fermentation.
(XLSX)

S2 File Raw file (*xlsx) for transcriptome of *P*. *expansum* 40815 in SmF and SSF. SmF: Submerged fermentation, SSF: Solid-state fermentation.
(XLSX)
