# Supplementary material for: Metabolomic and Transcriptomic Comparison of Solid-State and Submerged Fermentation of Penicillium expansum KACC 40815
Source: PLoS One. 2016 Feb 10;11(2):e0149012. doi: 10.1371/journal.pone.0149012 (PMC4749308; doi:10.1371/journal.pone.0149012)
Supplement: S1 Fig — SmF: Submerged fermentation, SSF: Solid-state fermentation. (PPTX) [file pone.0149012.s001.pptx]

## Slide 1
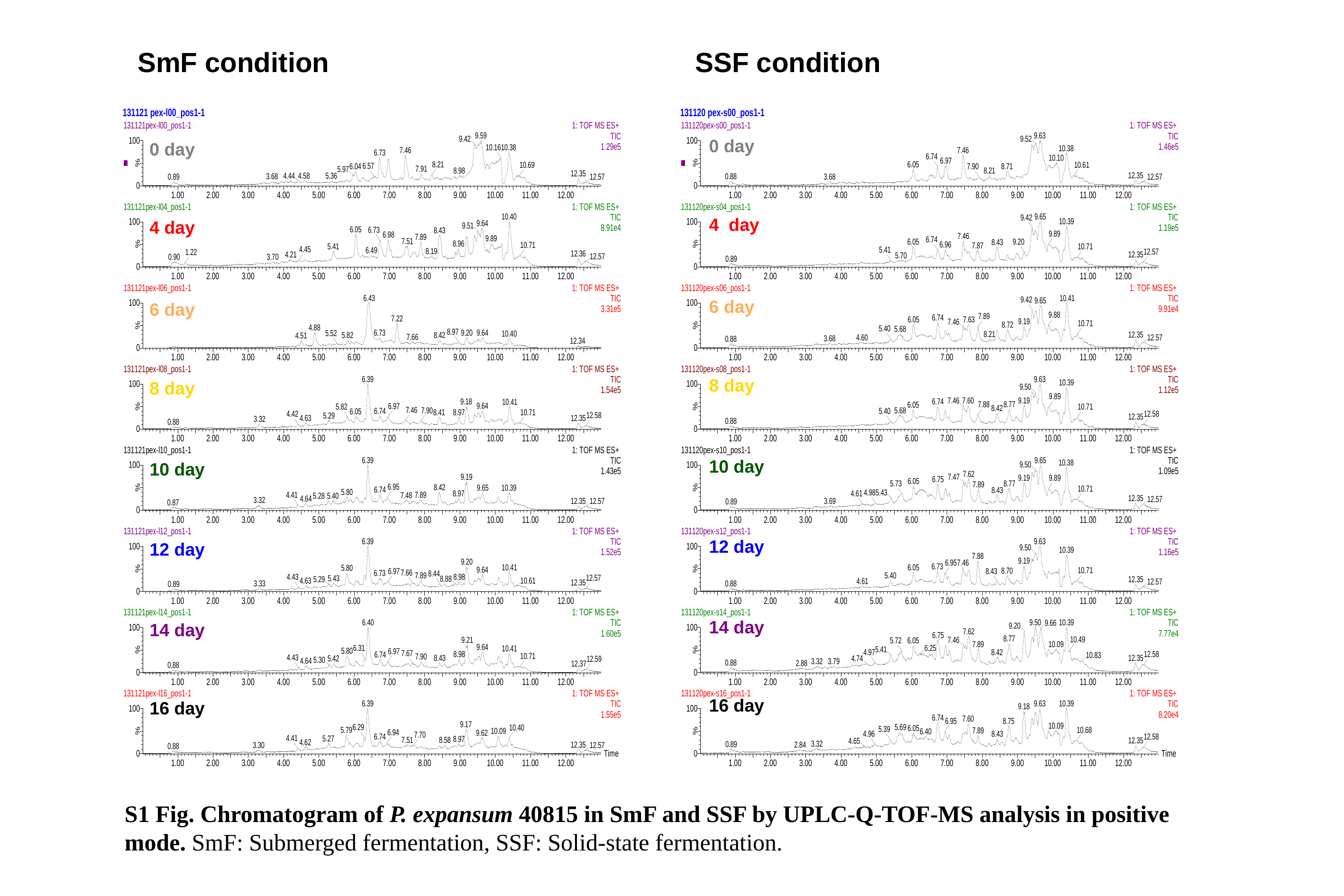

SmF condition
SSF condition
0 day
0 day
4 day
4 day
6 day
6 day
8 day
8 day
10 day
10 day
12 day
12 day
14 day
14 day
16 day
16 day
S1 Fig. Chromatogram of P. expansum 40815 in SmF and SSF by UPLC-Q-TOF-MS analysis in positive mode. SmF: Submerged fermentation, SSF: Solid-state fermentation.

## Slide 2
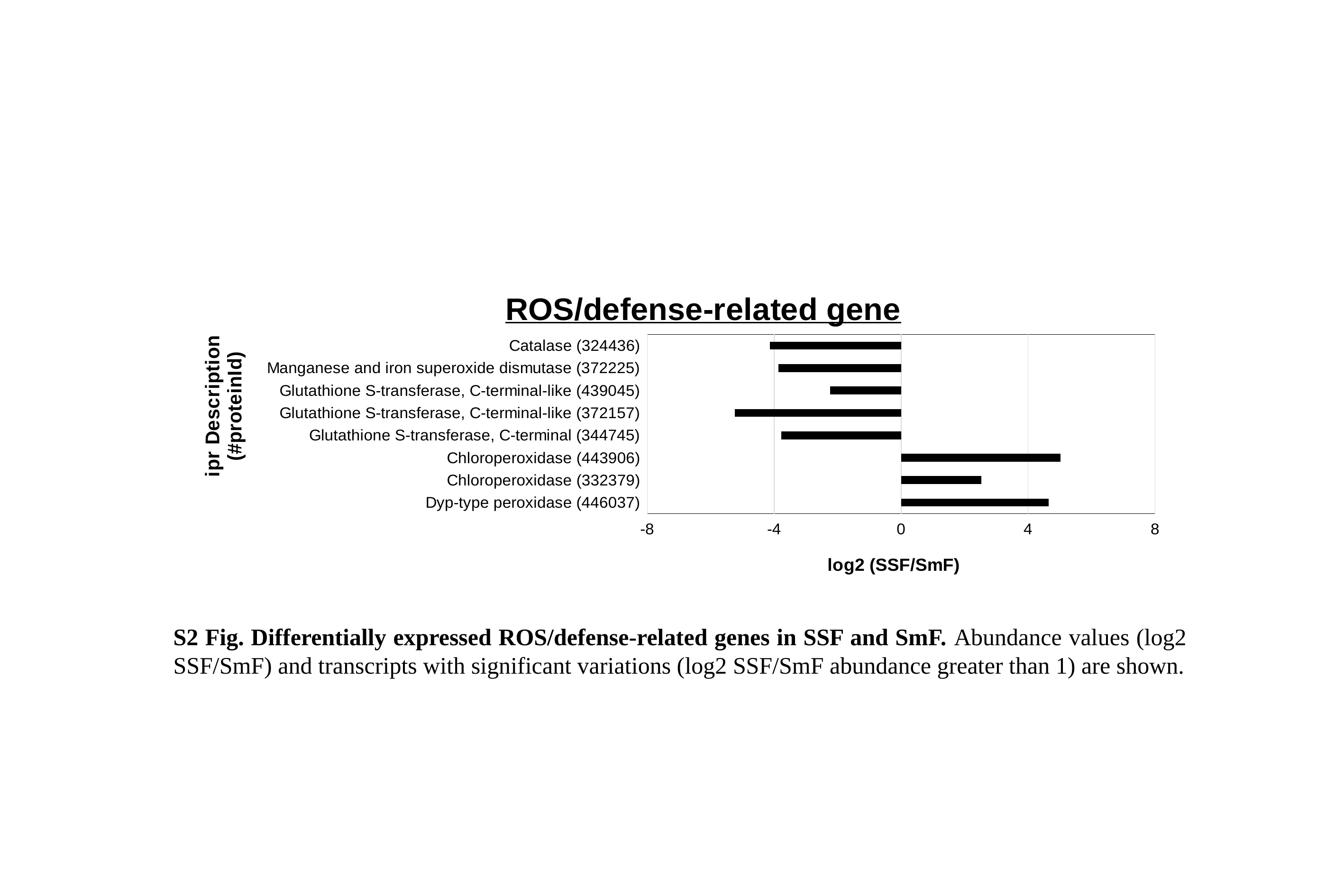

### Chart: ROS/defense-related gene
| Category | ROS/defese-related gene |
|---|---|
| Dyp-type peroxidase (446037) | 4.65237 |
| Chloroperoxidase (332379) | 2.52536 |
| Chloroperoxidase (443906) | 5.01722 |
| Glutathione S-transferase, C-terminal (344745) | -3.77391 |
| Glutathione S-transferase, C-terminal-like (372157) | -5.23707 |
| Glutathione S-transferase, C-terminal-like (439045) | -2.23502 |
| Manganese and iron superoxide dismutase (372225) | -3.86838 |
| Catalase (324436) | -4.13225 |S2 Fig.
S2 Fig. Differentially expressed ROS/defense-related genes in SSF and SmF. Abundance values (log2 SSF/SmF) and transcripts with significant variations (log2 SSF/SmF abundance greater than 1) are shown.

## Slide 3
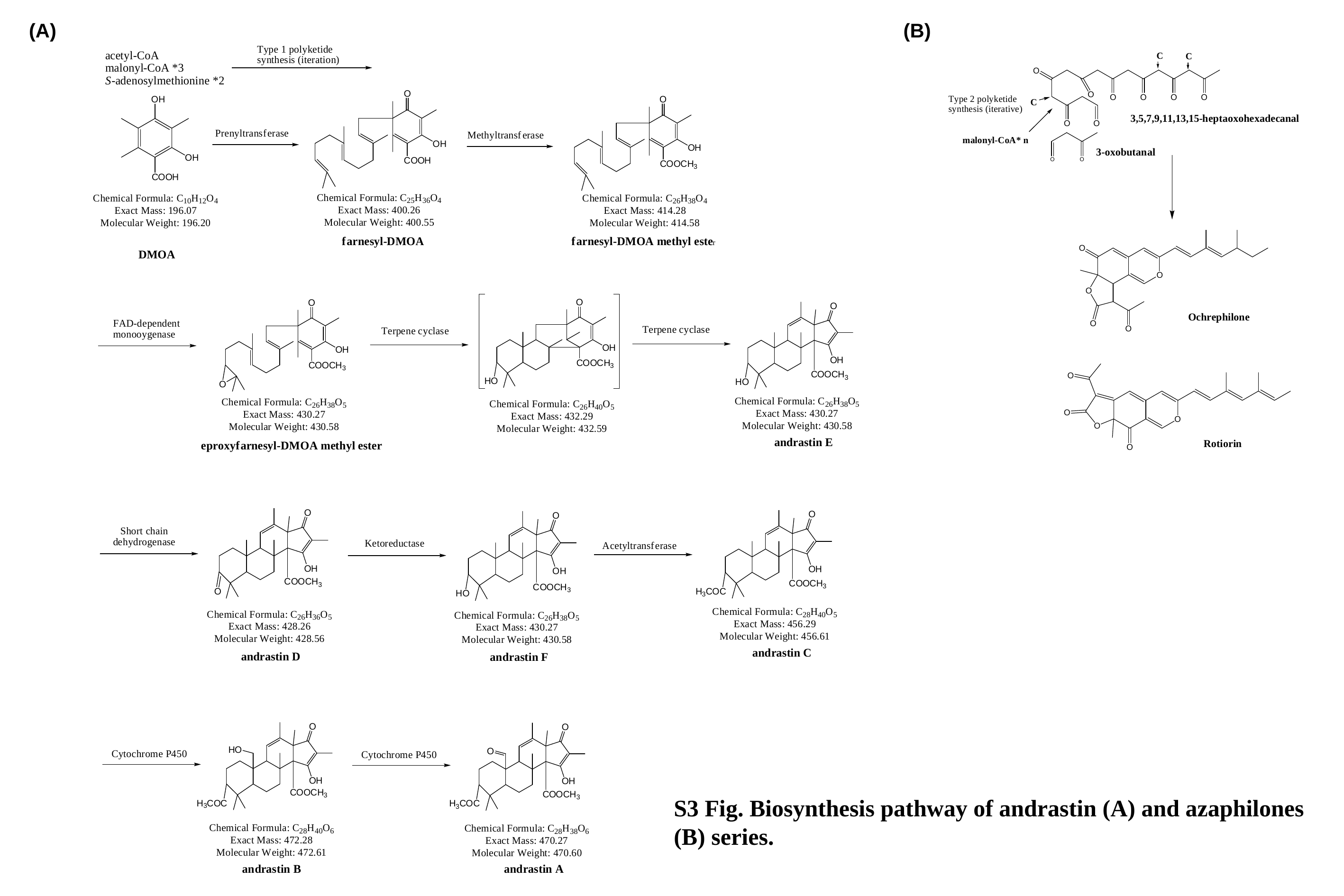

(A)
(B)
S3 Fig. Biosynthesis pathway of andrastin (A) and azaphilones (B) series.
