# Supplementary figures and images for: Metabolomic and Transcriptomic Comparison of Solid-State and Submerged Fermentation of Penicillium expansum KACC 40815
Source: PLoS One. 2016 Feb 10;11(2):e0149012. doi: 10.1371/journal.pone.0149012 (PMC4749308; doi:10.1371/journal.pone.0149012)

## Slide 1
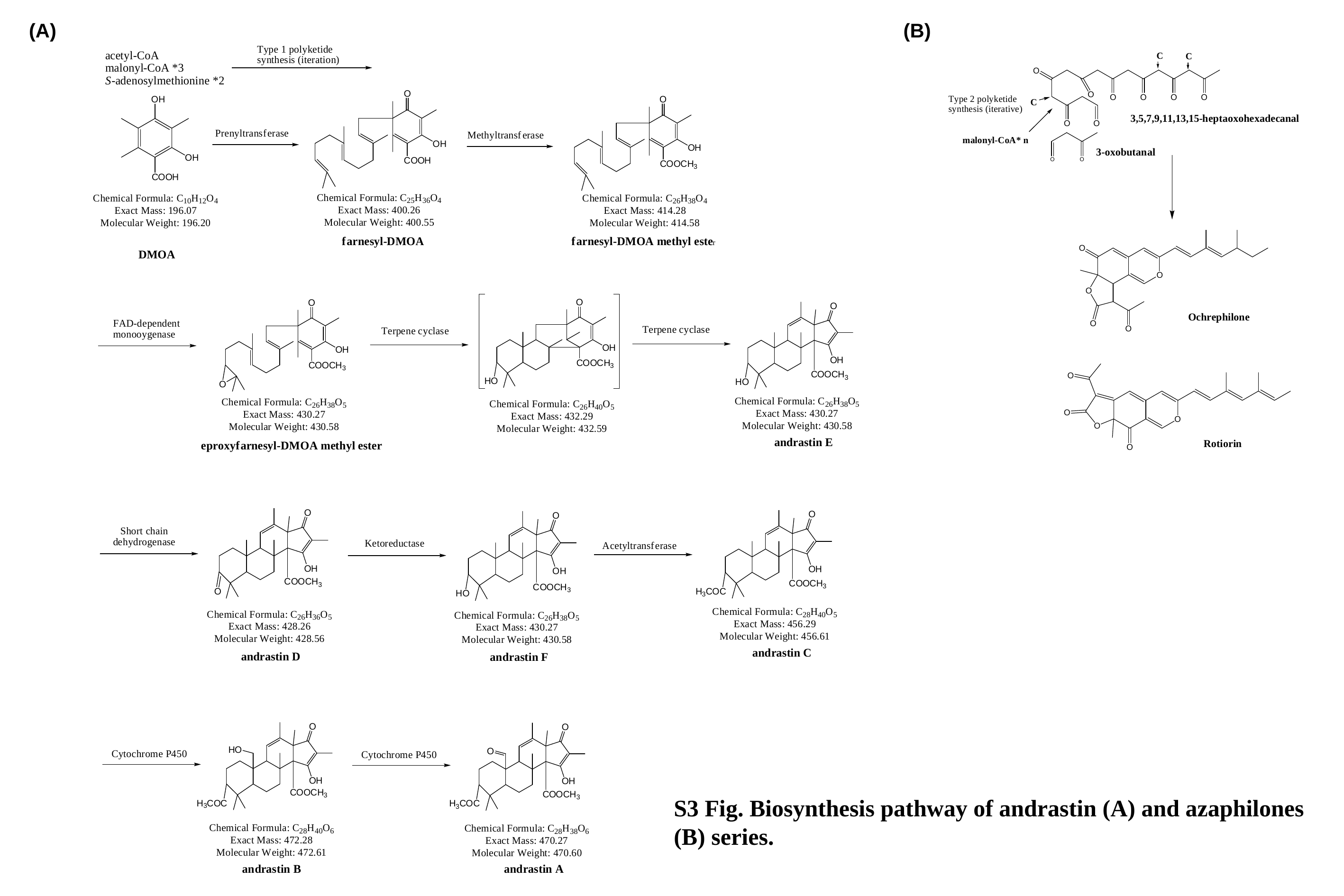

(A)
(B)
S3 Fig. Biosynthesis pathway of andrastin (A) and azaphilones (B) series.

Supplement: S3 Fig — (PPTX) [file pone.0149012.s003.pptx]
